# Polyurethane synthesis for vascular application

**DOI:** 10.1007/s40204-018-0101-6

**Published:** 2018-10-22

**Authors:** Zahra Zaredar, Fahimeh Askari, Parvin Shokrolahi

**Affiliations:** 1 Polymer Science Department, Iran Polymer and Petrochemical Institute, Tehran-Karaj Hwy, Tehran, 14977-13115 Iran; 2 Biomaterials Department, Iran Polymer and Petrochemical Institute, Tehran-Karaj Hwy, Tehran, 14977-13115 Iran

**Keywords:** Vascular applications, Polyurethane, POSS nano-particles, Siloxane macrodiol, Biocompatibility

## Abstract

**Abstract:**

Three polyurethane formulations were prepared on the basis of siloxane; two formulations contained 1% and 3% of a hydroxyl functionalized polyhedral oligomeric silsesquioxane [POSS (ROH)_2_] nano-particles (as a co-chain extender) and one was without nano-particle. Structures of the polyurethanes were characterized by FTIR and SEM. The effect of POSS nano-particles on properties of the synthesized PUs was examined for vascular applications by tensile test, contact angle, SEM, AFM and endothelial cells viability evaluation. Properties of the polyurethane with 1% POSS were compared with those of PU without POSS and the results showed 66% increase in the elongation-at-break, 53% increase in tensile strength and 33% increase in modulus, 9.45% increase in contact angle, 76.7% reduction in surface roughness and 9.46% increase in cell viability. It was also shown that a polyurethane containing 1% of POSS nano-particles in its structure developed the highest hydrophobicity, which resulted in its lowest potential for thrombosis.

**Graphical Abstract:**

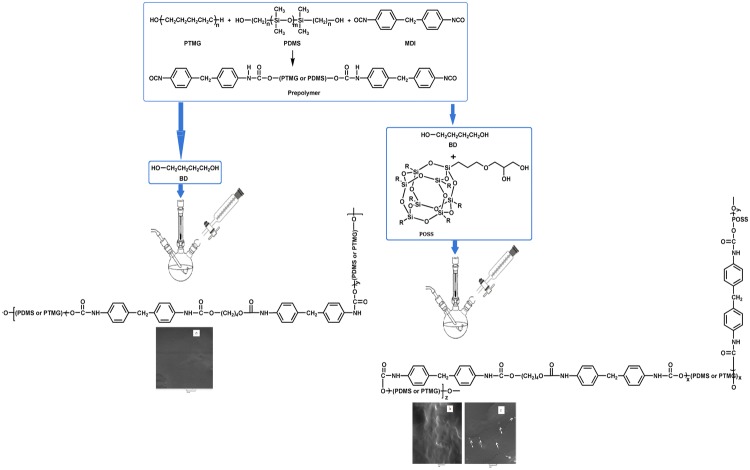

## Introduction

The synthesis of a suitable polymeric system in vascular applications is one of the vital issues that material engineers can help physicians and surgeons (Grundfest-Broniatowski 2013). Polyamide (nylon), polypropylene, polytetrafluoroethylene (Teflon), expanded polytetrafluoroethylene (ePTFE) and polyethylene terephthalate are the polymers that have been studied for their suitability in vascular applications (Prisacariu 2011).

Polyurethanes are a large family of polymers that have been introduced as biomaterials since Boretos and Pierce 1967. Since then, due to the desirable specifications such as biocompatibility, good hydrolytic properties, oxidative biostability, suitable mechanical properties, and good processability have been considered in medical applications (Boretos and Pierce 1967).

At present, expanded polytetrafluoroethylene (ePTFE) and dacron are used in the human body as two standard materials for production of a large-sized arterial vein with diameters > 6 mm, which both meet the requirements of those veins. However, for small size vessels with diameters < 6 mm, still autologous graft is the only approach available in clinics (Kapadia et al. 2008 and Ravi and Chaikof 2010; Ahmed et al. 2014).

Recently, researchers have developed polycarbonate-based polyurethane, included with polyhedral oligomeric silsesquioxane nano-particle (POSS-PCU) for application as small size artificial arteries. On in vivo evaluation, after 9 months of implantation in the animals, 64% of the cases were free from aneurysm and calcification (Ahmed et al. 2014).

Chemical structure of polyurethanes consists of soft and hard segmental domains. The soft segment is made of polyol, and the hard segment includes the isocyanate with chain extender used (alcohol or amine) in the synthesis. The soft segment forms the amorphous domain of the chains and is responsible for providing properties such as flexibility. The hard segment forms the crystalline domain of the chain, and the urethane (NHCOO) or urea (NHCONH) bonds occur in this domain. The determinations of glass transition temperature and thermal stability come from this domain (Szycher 2013; Thomson 2005; Barikani 2006).

Polyurethanes (PUs) were considered in this research work mainly because polymers can be tailor made to match the properties of vascular prosthesis. This means that PUs can provide different physical, mechanical and biological properties by aptly selection of the soft and the hard segment components, according to vascular implant requirements.

In this study, three different formulations of polyurethane were synthesized and their properties were investigated. In the synthesis of these polyurethanes, polydimethyl siloxane (PDMS), because of biocompatibility, high flexibility, very low surface energy (20 to 21 dynes/cm) and resistance to oxidative degradation (Askari 2013), was used as the soft domain. Considering poor mechanical properties of PDMS, in addition polytetramethylene ether glycol (PTMG) was used as a second macrodiol to compensate this disadvantage. In the synthesis of PUs, the POSS nano-particles were entered into the chain structure along with butanediol, as a co-chain extender, to investigate the effect of nano-particles on the synthesized PUs’ mechanical and biological properties.

## Materials

Polydimethylsiloxane (PDMS Mn 2500) (hydroxyl value 45 mg KOH/g) was purchased from Evonik Goldschmidt Co; 4,4ʹ-methylenediphenyl diisocyanate (MDI), polytetramethylene ether glycol (PTMG Mn 2000) (hydroxyl value 53–59 mg KOH/g), 1,4-butanediol (BD) was purchased from Sigma-Aldrich; tetrahydrofuran (THF), toluene was purchased from Merck CO; 1,2-propanediol isobutyl polyhedral oligomeric silsquioxane (POSS) was purchased from Hybrid Plastics Co. PDMS and PTMG were dried prior to use under vacuum at 80 °C for 24 h; BD was dried prior to use under vacuum at around 55 °C for 48 h.

### Synthesis of polyurethane

Polyurethane was synthesized by solution polymerization in two steps using a 250 mL round-bottom flask equipped with a mechanical mixer passed through a condenser. One flask neck was used for entering nitrogen gas and the other for input of materials. First, the MDI was introduced into the flask at 70–75 °C and melted. A solution of dry macrodiols with polydimethyl siloxane:polytetra methylene glycol at weight ratio of 8:2 as a soft segment in a 50:50 (w/w) mixture of tetrahydrofurane and dry toluene was prepared and added to the flask. Thus, a mixture with the ratio of 50:50 (w/w) of solids:solvent was made. After 45 min, the chain extender was added. BD was added as a chain extender in a polyurethane system without nano-particles, and in those PU setups containing nano-particles, POSS solution of tetrahydrofuran and BD were added together. After 45 min addition of chain extender, and ensuring completion of the process through FTIR test, a high viscous polymer was obtained and transferred into a Teflon mold.

### Characterization

Fourier transform infrared spectroscopy (FTIR) was used to illuminate the structure and chemical bonding. The test was carried out on a Bruker Equinox 55 FTIR spectrometer (Bruker, Germany) using films prepared from urethane copolymer and attenuated total reflection (ATR) equipped with Golden Gate single reflection at 4 cm^−1^ resolution.

The images from the surfaces of the samples, their fractured surfaces and the presence of the elements on the surfaces were prepared using a Tescan scanning electron microscope (SEM) (Vega series, Czech Republic). Before analysis, the specimens were placed on an aluminum base and coated with a gold coating using a sputter coater.

The atomic force microscope (AFM) test was carried out in AFM Dualscope/Rasterscope C26 (DME, Denmark) in noncontact mode. SPM-DME software was used for determining the roughness of the surfaces.

Mechanical properties of the synthesized polyurethanes were investigated by a Santam mechanical testing machine (STM-20, with a 60 N load cell, Iran), at a crosshead speed of 10 mm min^−1^ and gauge length of 18 mm, according to ASTM D 638. The samples were cut from hot-pressed film, in strips of 28 mm × 0.9 mm × 5 mm dimensions. The reported results are an average of 3–4 measurements (these tests were performed after ensuring complete removal of the solvent from the substance bed).

The hydrophilicity of the surface of the synthesized polyurethanes was investigated by measuring the contact angle of water droplet on the film surface. For this purpose, water droplets were placed on a sample surface using a micro-syringe and photographed. The contact angle of the specimens was determined using image-J software and tangent drawing. Four tests were performed and the mean of the angles was reported.

The cell viability and proliferation assay were carried out based on a colorimetric method in which methylthiazole tetrazolium (MTT) was reduced by a dihydrogenase enzyme in the cellular plasma, resulting in the formation of a purple product called formazan. The total amount of this purple product was determined by optical absorption, using a Sigma MTT kit for this test. The sterilized specimens with 70% ethanol were placed in cell culture plates each with 96 wells. In parallel, a number of wells were cultured with no sample and used as negative controls. After adding the culture medium and a definite number of cells into each well and at desired culture duration, the MTT (5 mg/mL) solution was added at a ratio of 1:5 and incubated at 37 °C for 2 h in 5% CO_2_ atmosphere to form purple formazan crystals. It should be noted that the above time is different for various cells. After that, the MTT solution was removed from the cells and 100 μL of isopropanol was added into each well of the cell culture plate and incubated for 1 h. Finally, the absorbance of the solution was measured and recorded using a microplate counter at 540 nm wavelength. The cellulose proliferation was calculated. Three samples were tested and the results were averaged.

Differential scanning calorimetry (DSC) was performed under nitrogen atmosphere on a Netzsch DSC 200 F (Netzsch, Bavaria, Germany) fitted with an air cooling compressor at a constant rate of 10 °C/min reciprocating from −130 to 250 °C and 250 to −130 °C and finally from −130 to 300 °C to eliminate thermal history.

## Results and discussion

### Synthesis of polyurethane

Polyurethanes were synthesized by a two-step polymerization method.

First, a pre-polymer was made by combining the molten MDI, with PDMS and PTMG mixed in a 1:1 (vol/vol) THF/toluene solvent system. The first PU was made using BDO as the sole chain extender (PU-BDO), while in two other samples, BDO and POSS were added as co-chain extenders (PU-BDO/POSS).

**Fig. 1:**
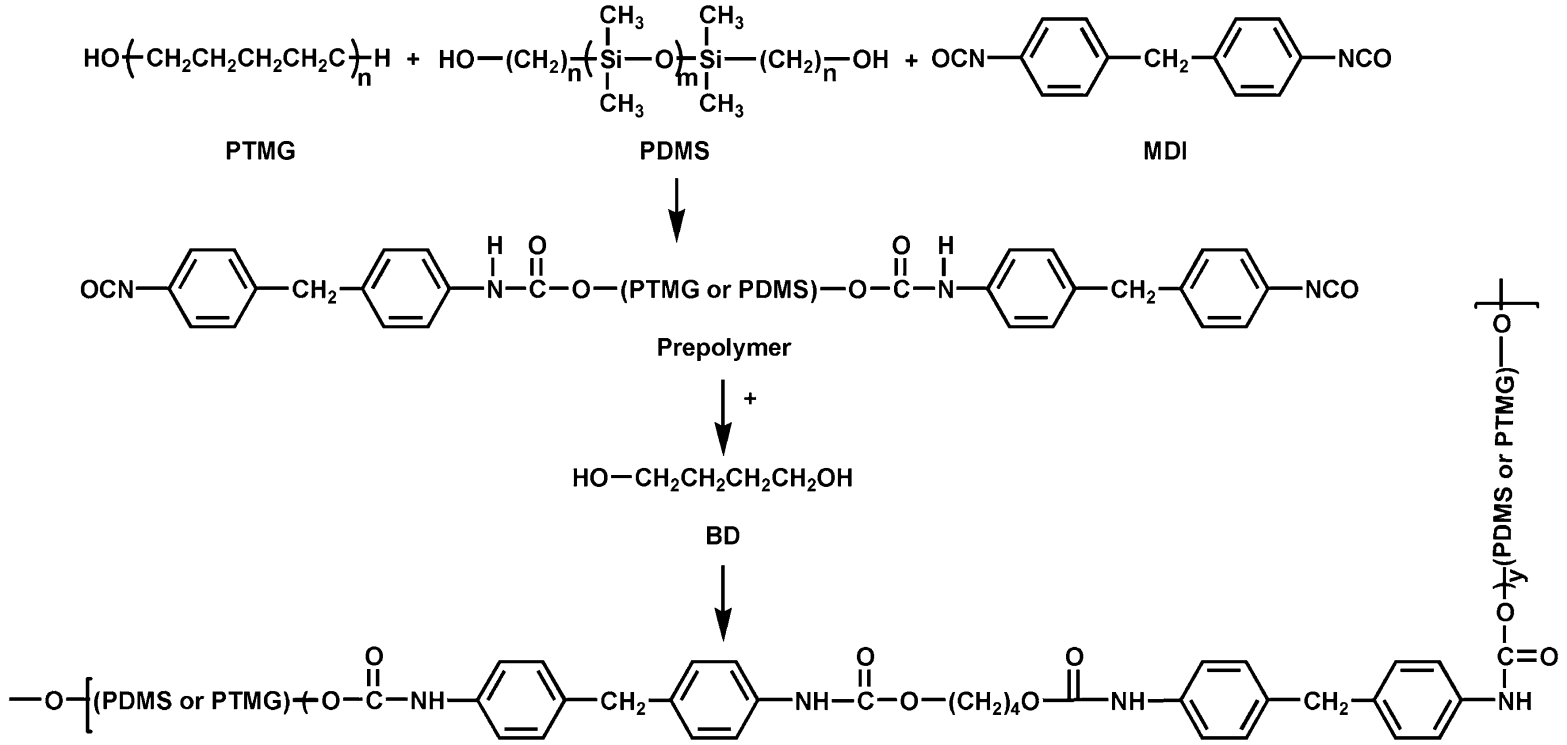
Synthesis route of PU-BDO

**Fig. 2:**
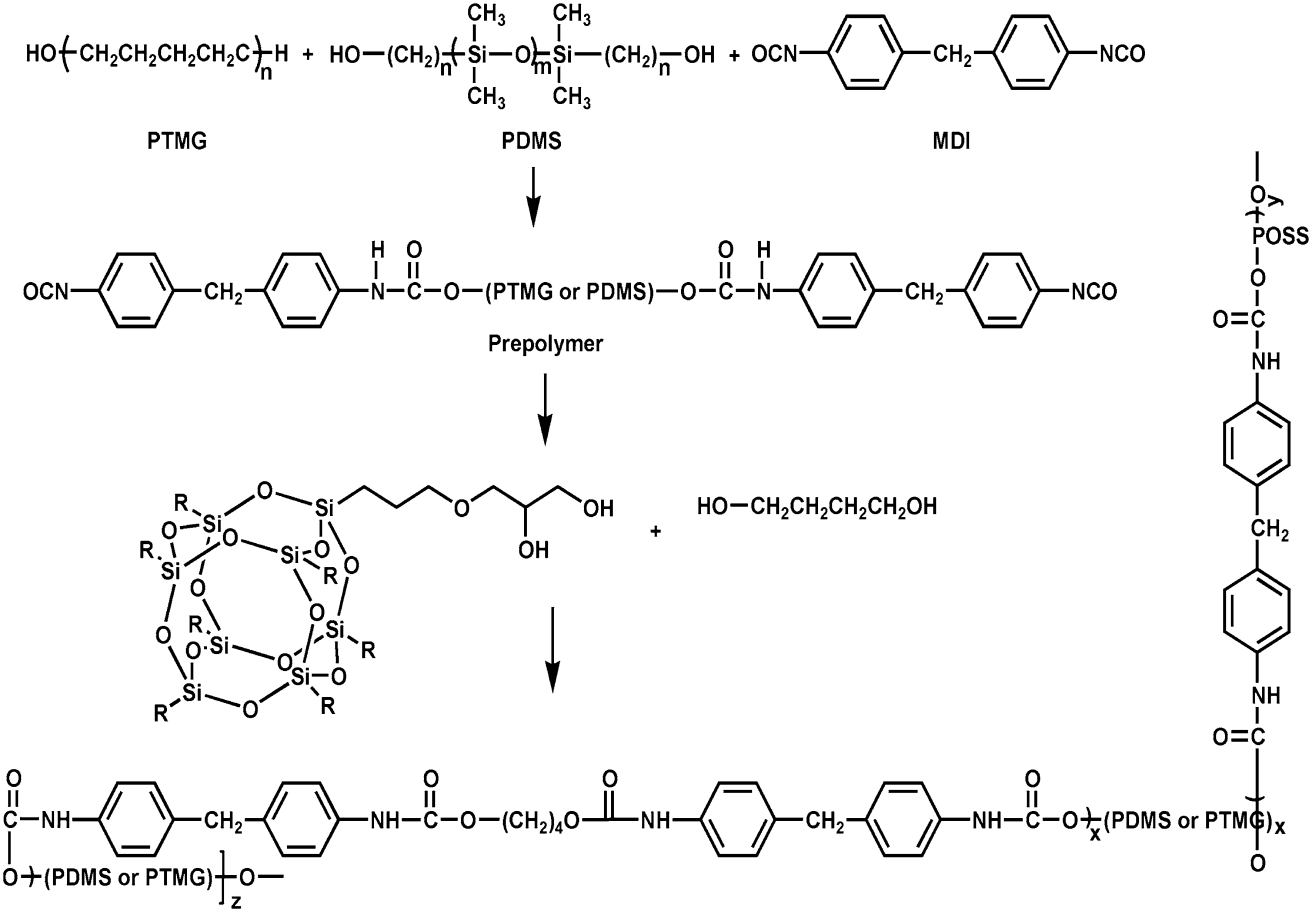
Synthesis route of PU-BDO/POSS

Synthetic routes of PU-BDO and PU-BDO/POSS are displayed in Figs. 1 and 2, respectively.

**Fig. 3:**
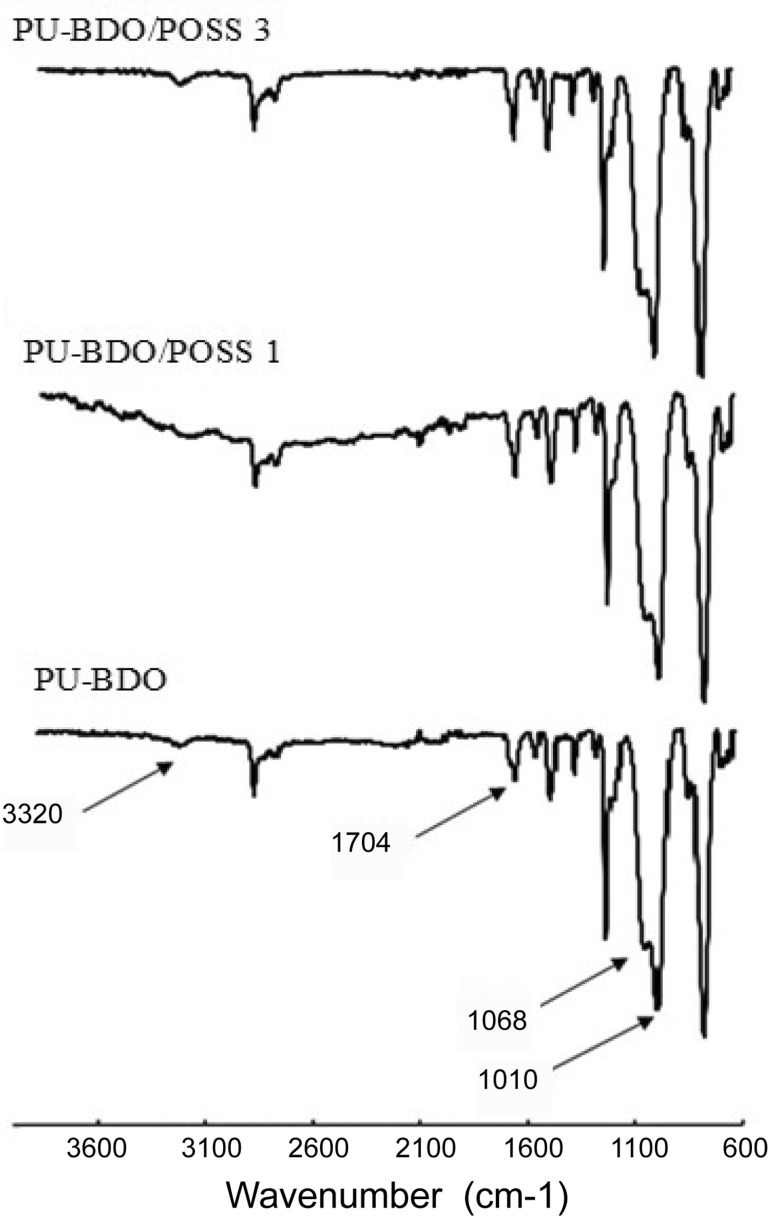
ATR-FTIR spectra of the PUs

ATR was used to confirm the formation of polyurethane structure. The synthesized polyurethanes with and without nano-particles have similar ATR spectra (Fig. 3). Disappearance of the peak at 2270 cm^−1^ indicates that the isocyanate groups were completely consumed in the synthesis and no free isocyanate was remained. Also, appearance of the peaks at 3220 cm^−1^ and 1704 cm^−1^ confirms the formation of urethane bonds. Other bonds in the chemical structure of the synthesized polyurethanes are shown in Table 1. Our results are in agreement with those previously reported by Bai et al. (2008; Zia et al. 2014).

**Table 1:** Characteristic FTIR peak specifications of PUs (Bai et al. 2008; Zia et al. 2014)

Links	Wavelength (cm^−1^)
Stretching vibrating SiOSi bonds	1010, 1068
C=O (urethane, H-bonded)	1704
Vintage vibrational integration of NH urethane and OH	3320
Stretching vibrational CH in SiCH_3_	1257
The bending vibration H connected to C=C	730
Stretching vibrational C=C in aromatic ring	1675
Stretching vibrational CH and CH_2_	2955
CH_2_ bending vibration CH	1457
Bending vibration CH_3_	2850
Bending vibration CH_3_	1365

### Scanning electron microscopy

**Fig. 4:**
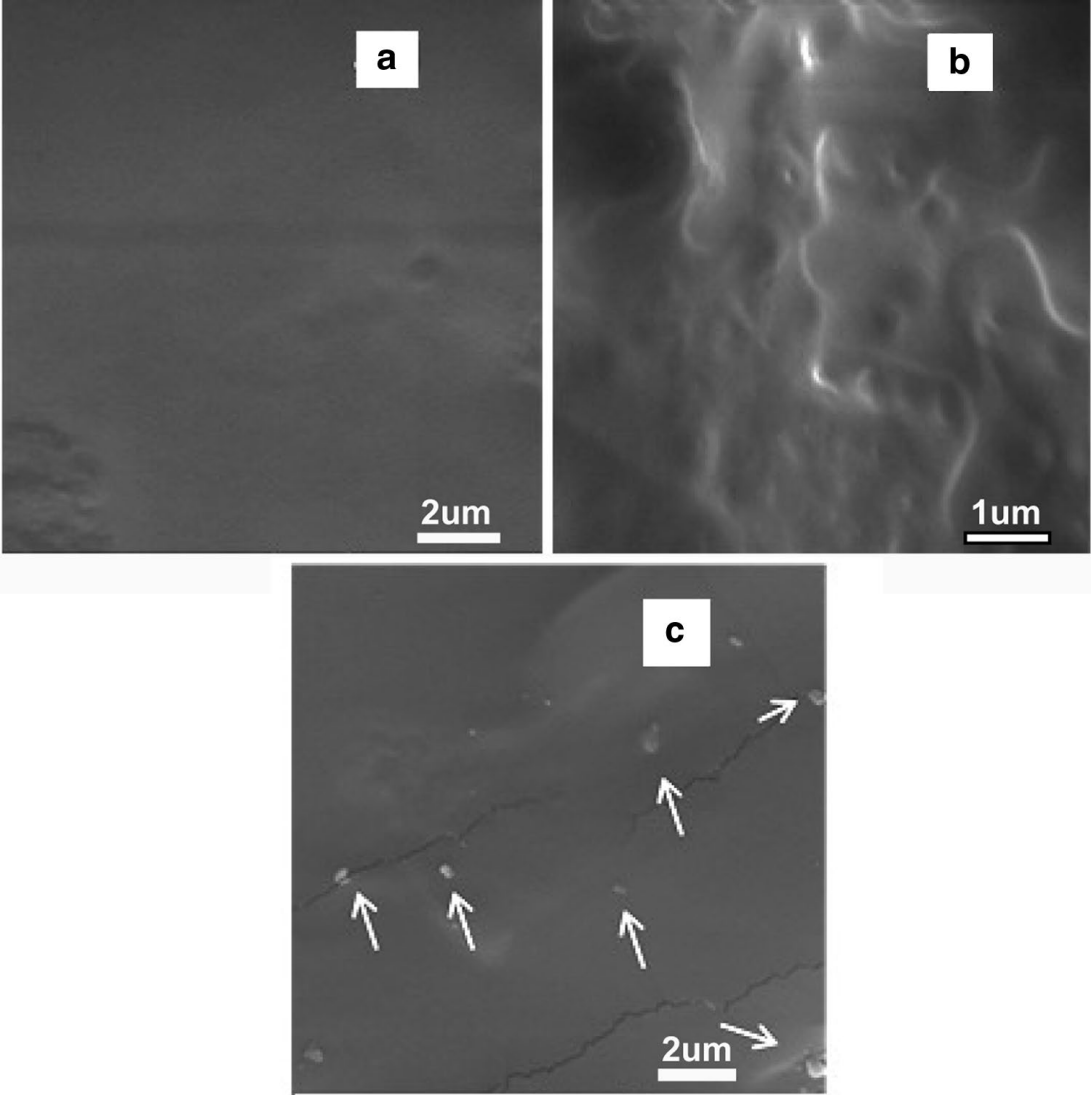
SEM images taken at the PUs’ fracture surface; **a** distribution of nano-particles in PU-BDO, **b** distribution of nano-particles in PU-BDO/POSS 1, and **c** distribution of nano-particles in PU-BDO/POSS 3

Microstructures of the synthesized polyurethanes were studied by scanning electron microscope (SEM). The polyurethane samples were prepared through fracturing in liquid nitrogen and their fracture surface was sputter coated with a thin layer of gold and studied with SEM. Figure 4a–c shows the fracture surface of the polyurethane samples without POSS (PU-BDO), and with 1% and 3% POSS (PU-BDO/POSS 1 and PU-BDO/POSS 3), respectively. As the nano-particles are incorporated as co-chain extender in this study (Fig. 4b), in PU-BDO/POSS1 they are well integrated into the polyurethane structure. This means that the nano-particles have successfully entered into PU chain and played the role of chain extender well. Nevertheless, as it is depicted in Fig. 4c, as related to PU-BDO/POSS3, it is observed that some nano-particles have been aggregated and agglomerated (arrows in Fig. 4c).

### Mechanical properties

**Fig. 5:**
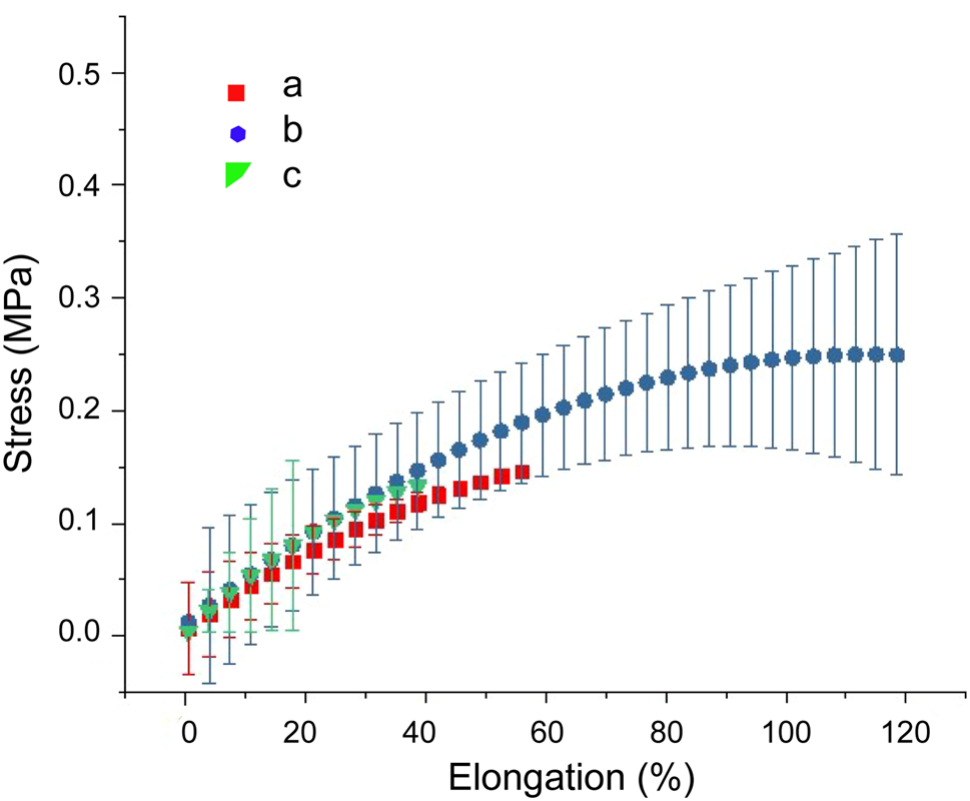
Stress–stress curves of the synthesized PU-BDO, PU-BDO/POSS 1, and PU-BDO/POSS 3

Mechanical properties of the synthesized polyurethanes were measured in tensile mode and the stress–strain curves acquired for PU-BDO, PU-BDO/POSS1, and PU-BDO/POSS3 are presented in Fig. 5a–c, respectively. As shown in Fig. 5, the addition of 1% nano-particles caused 66% increase in the elongation-at-break and 53% increase in the tensile strength. Also, the tensile modulus value has increased by 33%. But by further increase in the amount of nano-particles from 1 to 3%, although maintaining a 33% increase in modulus, it has led to loss of strength and elongation-at-break. The values of elongation-at-break, strength, and Young modulus are reported in Table 2.

**Table 2:** Mechanical properties of the synthesized polyurethanes

Samples	Modulus (kPa)	Strength at-break (kPa)	Strain-at-break (%)
PU-BDO	0 ± 3	0.04 ± 150	11 ± 60
PU-BDO/POSS 1	0 ± 4	0.03 ± 230	7 ± 126
PU-BDO/POSS 3	0 ± 4	0.02 ± 120	6 ± 35

Various studies, including that by Teng and Qiu research in 2016, demonstrated that the addition of a small amount of POSS has led to mechanical properties’ improvement. Teng and Qiu 2017 introduced 0.5 wt% and 1 wt% of POSS into polyethylene succinate structure using a common solvent between nano-particles and polyethylene succinate. The result indicated that the addition of 0.5% and 1% POSS to the base polymer increased the modulus, strength and elongation-at-break of the PUs compared to that without nano-particles. By addition of 0.5% nano-particles, there was greater improvement in the strength and elongation-at-break, compared to that of 1% nano-particles (Teng and Qiu 2017).

The reason behind the observed increase in the mechanical properties of PU-BDO/POSS1 and decrease in the mechanical properties of PU-BDO/POSS3, both compared with that of PU-BDO, can be attributed to the different microstructures of these systems. As shown in Fig. 4, in PU-BDO/POSS 1, nano-particles are uniformly entered in the structure of the chains, while in PU-BDO/POSS 3, it is observed the POSS nano-particles did not fully integrate into the structure of the chains, which caused sample to have points for stress concentration and, thus, the sample breaks at smaller stress and strain than PU-BDO/POSS 3.

### Contact angle

The water–biomaterial contact angle is an important criterion in the determination of the efficacy of implants in contact with blood. Hydrophobicity enhancement decreases the platelet adhesion and also protein uptake (Kidane et al. 2009). Reducing protein uptake is important because absorption of blood plasma protein is one of the first problems that very often occurs after prosthesis implantation and leads to thrombosis, as well as inflammatory responses and cellular interactions. Release of various enzymes and water-soluble agents, including growth factors or cytokines, are of cell inflammatory responses that lead to destruction and ultimate failure of the implant (Cassim et al. 2002; Azevedo and Reis 2005; Christenson et al. 2006).

It is also important to note that when a vascular prosthesis is implanted in the body, it is expected that the endothelial cells grow and proliferate on its surface. For such an endothelial behavior, a contact angle of about 55° revealed the best performance (Wang et al. 2010; Solouk et al. 2011).

In this research, water contact angle on the synthesized polyurethanes was measured by contact angle measurement of the water droplet with the PU films surfaces. Images of the droplets placed on the surface of the samples and the results are presented in Table 3.

**Table 3:**
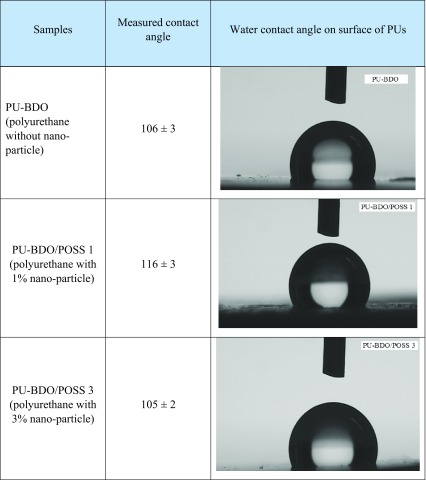
Contact angle of the polyurethanes

A contact angle of 106° was recorded for PU-BDO, and by the addition of the POSS nano-structures, as a result of the hydrophobicity of the substance, this value has been dropped. As the nano-structures used in this study are of a silicon nature and silicon is a hydrophobic substance (Wang et al. 2010), therefore, it is expected that the addition of POSS nano-particles leads to an increase in the material hydrophobicity. According to our expectation, with addition of just 1% POSS nano-particles, the water contact angle on the surface of PU-BDO/POSS1 has increased to 116. Similar behavior was previously reported by Seifaliyan et al. in 2009 (Kidane et al. 2009). They compared polyurethanes without and with 2% POSS nano-particles and reported on the tangible effect of POSS nano-particles on water contact angle.

Meanwhile, our results showed that by increase in loading of the POSS nano-particles to 3%, the contact angle dropped to 105°, which is approximately the same as that recorded for PU-BDO. Since contact angle is greatly influenced by surface roughness, AFM microscopy was carried out next to monitoring the possible variations in the surface function of the PUs as a function of POSS loading.

### Atomic force microscopy

**Fig. 6:**
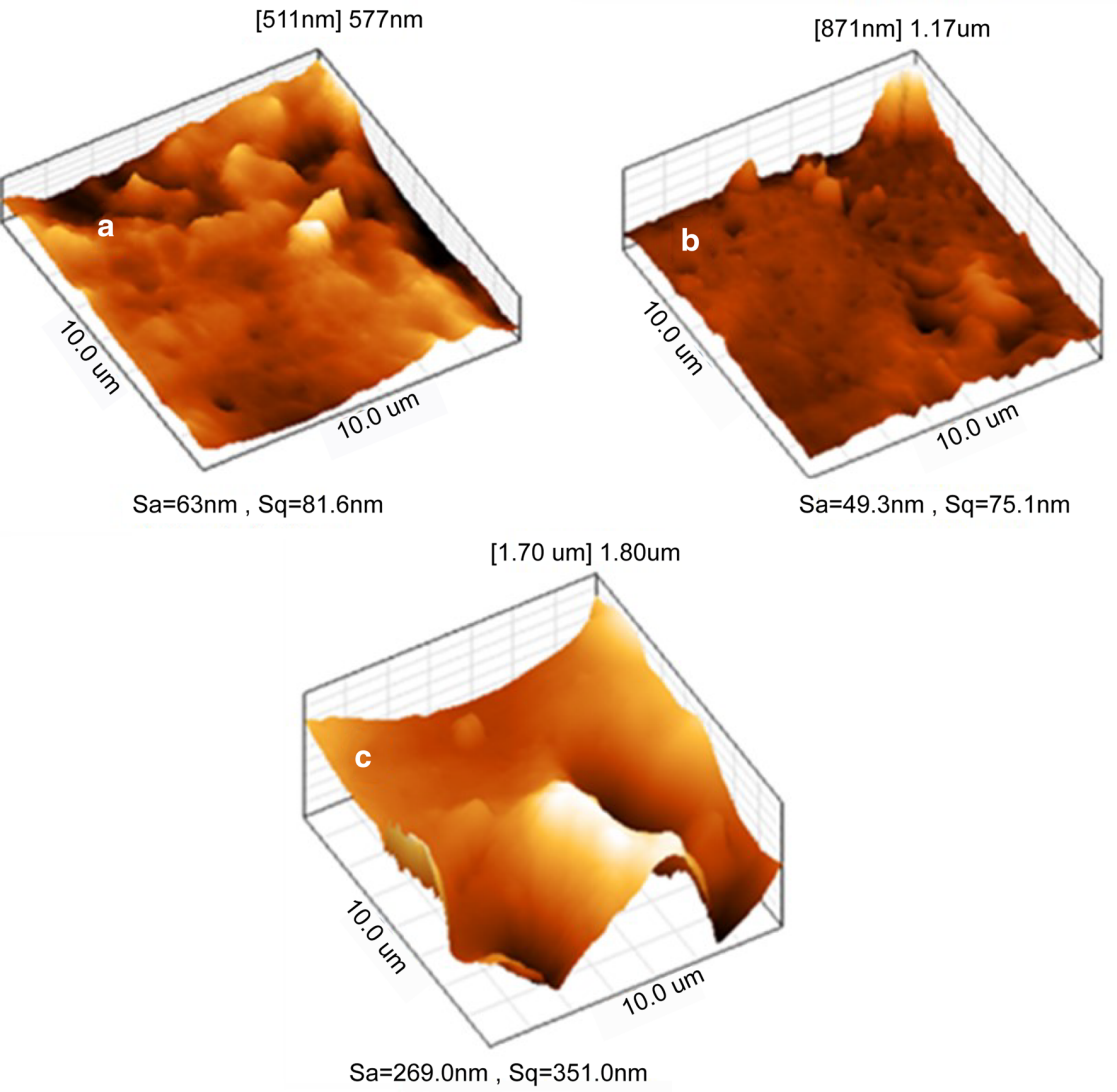
AFM images of the synthesized polyurethanes; **a** PU-BDO, **b** PU-BDO/POSS1, and **c** PU-BDO3

The roughness parameters were estimated by surface topography studies using AFM microscopy. AFM images of the PU-BDO, PU-BDO/POSS 1, and PU-BDO/POSS 3 in topographic mode and the measured values of the mean transverse profile height, expressing roughness (Sa), and mean square root, representing undesirable deviations (Sq) are presented in Fig. 6. As shown in Fig. 6, with the addition of 1% POSS nano-particles into siloxane-based polyurethane, the surface roughness is reduced. Meanwhile, by increasing the nano-particles content from 1 to 3%, surface roughness has increased significantly. The initial decrease in the surface roughness of PU-BDO, by the addition of 1% POSS, must be due to the fact that with introduction of POSS nano-particles into the PU-BDO polyurethane chain, and due to the silicon rich pendant groups of POSS, on their surfaces and low energy level, these silicon groups are arranged at the sample surface (Askari 2013) and fill the deformations on polyurethane surfaces.

This arrangement in the surface is also confirmed by the results obtained from the contact angle measurement. As shown in Table 3, the sample containing 1% nano-particles (PU-BDO/POSS 1) is more hydrophobic at surface compared to the nano-particle-free polyurethane (PU-BDO). Also it is seen that by increasing the nano-particle content from 1 to 3%, the roughness increases and the degree of hydrophobicity (contact angel) increased and becomes approximately equal to that of PU-BDO. The increase in roughness indicates that the loading amount of nano-particles is greater than the amount which could enter the chain structure as a chain extender. As a result, part of the nano-particle content remained unbounded in the form of aggregates within the PU matrix and outside the PU main chains.

In the SEM image (Fig. 4), these aggregated particles are visible. The observed increases in the surface roughness of PU-BDO/POSS 3 have lowered its corresponding contact angles (therefore, hydrophobicity has been reduced). Surface roughness and hydrophobicity are two critical issues regarding the use of artificial arteries. Considering that for application as artificial blood vessels, especially small artificial vessels, due to expected prolonged contact with blood (Ravi and Chaikof 2010), occurrence of thrombosis is the most important challenge (Solouk et al. 2015; Ahmed et al. 2014). A fact is generally accepted that thrombosis requires accumulation of platelets to occur on the artificial vein wall; the smaller the roughness of the surface (higher hydrophobicity), the lower the risk of thrombosis, and the material will be a better choice for the application as artificial artery. The results of topographic and contact angle showed that the addition of POSS nano-structures at a desirable loading amount of 1% to siloxane-based polyurethanes could satisfy both of these two important criteria.

### Toxicity

**Fig. 7:**
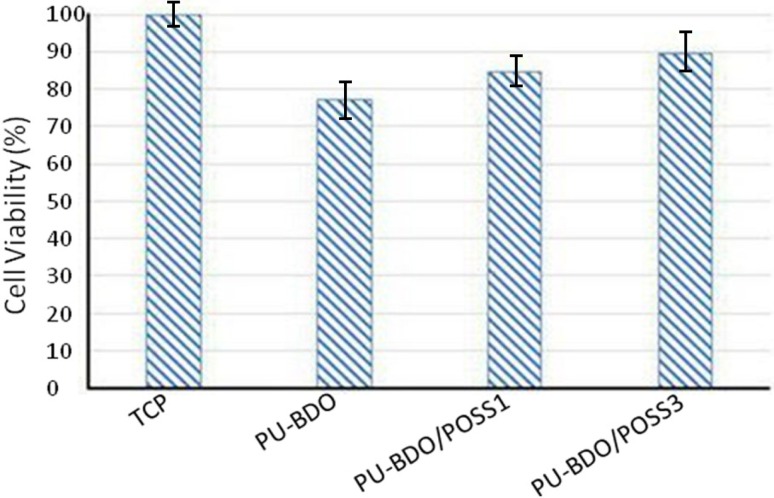
Cell viability evaluation result as measured by MTT assay. Standard deviations are less than 0.025 for all the samples

Since non-toxicity against cells is the first requirement of any medical implant (Grundfest-Broniatowski 2013) and due to the non-toxic surface of the POSS nano-particles (Kannan et al. 2006), it is expected that the presence of these particles in the structure should not show any undesirable effect on cytotoxicity of the synthesized polyurethanes. Biocompatibility enhancement of polyurethanes through introduction of POSS nano-structures into the polymer backbone was also observed by Seifalian et al. (Kidane et al. 2009). In the present study, MTT assay was used to investigate the effect of POSS nano-particles on biocompatibility of PU-BDO, PU-BDO/POSS 1 and PU-BDO/POSS 3. As shown in Fig. 7, an increase in the amount of nano-particles increased the biocompatibility and viability of L929 mouse fibroblasts. Figure 7 and Table 4 show viability of the cells on the surface of polyurethanes in comparison with that of the control (tissue culture plate, TCP).

**Table 4:** MTT test results

Polyurethanes	Viability (%)
PU-BDO	77
PU-BDO/POSS 1	85
PU-BDO/POSS 3	90

### Differential scanning calorimetry (DSC)

According to DSC analysis, PDMS polyurethane shows a glass transition at about − 116 °C that is related to PDMS soft segment. It is noted that the *T*_g_ of the soft segment did not change on addition of POSS nano-particles because the nano-particles were mainly involved in the hard segments. Also as shown in Table 5, melting point of the hard segment in polyurethane and nanocomposites is about 149 °C. According to the DSC results, introduction of the POSS cage in the hard segments of the PU resulted in enhancement in both the melting and crystallization enthalpies (slightly higher values were recorded for the POSS1 compared to those of the POSS 3).

**Table 5:** DSC results

Polyurethane	*T*_m_ (°C)	∆*H*_m_ (mJ)	*T*_c_ (°C)	∆*H*_c_ (mJ)
PU-BDO	149	− 11	92	33
PU-BDO/POSS 1	147	− 48	75	47
PU-BDO/POSS 3	149	− 43	84	41

*T*_m_: Hard segment melting point, ∆*H*_m_: Melting enthalpies of the hard segment, *T*_C_: Crystallization temperature of the hard segment, ∆*H*_C_: Crystallization enthalpies of the hard segment

## Conclusion

Considering the characteristics required for medical applications, the series of polyurethanes based on PDMS (80%) and PTMG (20%) were synthesized by solution polymerization using a mixture of THF and toluene solvents, and BD and POSS as chain extenders. Polyurethane formation was confirmed by ATR-FTIR spectroscopy. Mechanical properties’ measurement in tensile mode was performed to determine the effect of POSS nano-structures on the mechanical properties of the synthesized polyurethanes. Results of the mechanical properties measurement in tensile mode showed that using 1% POSS nano-particles resulted in 66% increase in the elongation-at-break, 53% increase in the strength, and 33% increase in the modulus. Further increase of the nano-particles up to 3%, although it may result in 33% increase in the modulus compared to POSS free polyurethane, but it has resulted in remarkable decrease in both strength and elongation-at-break compared to PU-BDO. To investigate the effect of nano-particles introduced in the material structure on cell viability, MTT test was performed and it was observed that the higher the percentage of added nano-particles, the better the cell viability.

It was also shown that a polyurethane, containing 1% of POSS nano-particles in its structure, reveals the highest hydrophobicity and in having the least potential for thrombosis and destruction.

Since surface roughness has a significant effect on platelet adhesion onto polymer surface, in investigating the platelet adhesion the surface roughness was studied by AFM and it was found that the sample containing 1% POSS nano-particle was less rough at surface compared to both the POSS-free sample and the sample with 3 wt% nano-particles. As a result, the sample containing 1% POSS nano-particles displays lower potential for platelet adhesion than that with 3 wt%. According to our results, the polyurethane containing 1% nano-POSS, with the highest mechanical properties, and water contact angel is a promising candidate as a blood compatible for application as vascular prosthesis.
